# Could gestational diabetes mellitus be managed through dietary bioactive compounds? Current knowledge and future perspectives

**DOI:** 10.1017/S0007114516000222

**Published:** 2016-02-16

**Authors:** Carmela Santangelo, Alessandra Zicari, Elisabetta Mandosi, Beatrice Scazzocchio, Emanuela Mari, Susanna Morano, Roberta Masella

**Affiliations:** 1 Department of Veterinary Public Health and Food Safety, Unit of Nutrition, Italian National Institute of Health, Viale Regina Elena 299, 00161 Rome, Italy; 2 Department of Experimental Medicine, 2nd Section of Cell Pathology, Sapienza University of Rome, Viale Regina Elena 324, 00161 Rome, Italy; 3 Department of Experimental Medicine, Section of Medical Pathophysiology, Food Science and Endocrinology, Sapienza University of Rome, Viale Regina Elena 324, 00161 Rome, Italy

**Keywords:** Gestational diabetes mellitus, Mediterranean diet, Dietary polyphenols, Adipokines, Molecular mechanisms, PUFA, MicroRNA

## Abstract

Gestational diabetes mellitus (GDM) is a serious problem growing worldwide that needs to be addressed with urgency in consideration of the resulting severe complications for both mother and fetus. Growing evidence indicates that a healthy diet rich in fruit, vegetables, nuts, extra-virgin olive oil and fish has beneficial effects in both the prevention and management of several human diseases and metabolic disorders. In this review, we discuss the latest data concerning the effects of dietary bioactive compounds such as polyphenols and PUFA on the molecular mechanisms regulating glucose homoeostasis. Several studies, mostly based on *in vitro* and animal models, indicate that dietary polyphenols, mainly flavonoids, positively modulate the insulin signalling pathway by attenuating hyperglycaemia and insulin resistance, reducing inflammatory adipokines, and modifying microRNA (miRNA) profiles. Very few data about the influence of dietary exposure on GDM outcomes are available, although this approach deserves careful consideration. Further investigation, which includes exploring the ‘omics’ world, is needed to better understand the complex interaction between dietary compounds and GDM.

Gestational diabetes mellitus (GDM) is the most common metabolic disorder during pregnancy^(^ ^1^ ^)^, and its prevalence is increasing worldwide^(^ ^2^ ^)^. Women with GDM are at a high risk of developing type 2 diabetes (T2D) later in life^(^ ^1^ ^)^; in addition, the higher baseline BMI and weight gain often found after GDM occurrence increase the risk of progression from GDM to T2D^(^ ^3^ ^)^. Moreover, uncontrolled GDM is associated with a detrimental intra-uterine environment, which leads to fetal complications and an increased risk for the child of developing obesity and metabolic disorders^(^ ^4^ ^,^ ^5^ ^)^.

In response to the marked rise in GDM, it is of paramount importance to identify appropriate treatments to prevent maternal and fetal complications associated with this disease.

At present, the management of GDM is a big challenge because of its heterogeneity (i.e. ethnic as well as intra-, and inter-country differences)^(^ ^2^ ^,^ ^6^ ^,^ ^7^ ^)^, and the incomplete knowledge of its pathophysiology^(^ ^8^ ^)^. As a result, standardised guidelines for GDM are difficult to arrive at worldwide, as are the intervention strategies aimed at preventing or/and reducing the burden of this disorder. In addition, obesity and maternal overweight have been considered as the main risk factors for GDM^(^ ^1^ ^)^; the reduction of these risk factors is essential for the well-being of mother and offspring^(^ ^5^ ^)^.

Pre-pregnancy and early pregnancy are the best periods for a dietary intervention to control weight in order to prevent the long-lasting effects of maternal diabetes or obesity^(^ ^9^ ^)^.

There is increasing evidence that the dietary patterns having beneficial effects both in prevention and management of diabetes are characterised by high consumption of plant foods (e.g. whole grains, fruit, vegetables and extra-virgin olive oil, nuts) and fish, and low consumption of animal-based, high-fat, processed foods, that is, the Mediterranean-style diet (MedDiet)^(^ ^10^ ^)^. The MedDiet is a primarily plant-based dietary pattern that has been strongly associated with lower incidence of CVD, and neoplastic diseases, and an overall reduced mortality^(^ ^11^ ^,^ ^12^ ^)^. Adherence to the MedDiet correlates with better glycaemic control, a reduced risk of both total and cardiovascular mortality in diabetic subjects in Mediterranean populations^(^ ^13^ ^–^ ^15^ ^)^ and a lower risk of metabolic syndrome and CVD in non-Mediterranean populations^(^ ^16^ ^–^ ^19^ ^)^. These findings indicate that the adoption of the MedDiet model by populations having different dietary habits is effective in reducing the risk of non-communicable disorders.

The short-term – 4 weeks – consumption of a DASH diet (Dietary Approaches to Stop Hypertension), rich in fruits, vegetables, whole grains and lower amounts of SFA, improved pregnancy outcomes among GDM women^(^ ^20^ ^)^. Pre-pregnancy adherence to healthy dietary patterns^(^ ^21^ ^)^ and the MedDiet is associated with lower incidence of GDM and a better degree of glucose tolerance in no-GDM pregnant women^(^ ^22^ ^)^.

In any case, the mechanisms underlying the protective effects of the MedDiet are not clear as yet. The high MUFA:SFA ratio, the increased level of PUFA, the low content of *trans*-fatty acids (FA) and the high content of fibres, vitamins, mineral salts and phytochemicals compounds may contribute to the beneficial effects of MedDiet^(^ ^23^ ^–^ ^25^ ^)^. Over the past years, researchers have focused their attention on the role of plant-derived, functional foods and their bioactive compounds in the control of various aspects of diabetes mellitus^(^ ^26^ ^,^ ^27^ ^)^. Among the known natural bioactive components, polyphenols have been shown to have anti-hyperglycaemic effects, antioxidant and anti-inflammatory activities and no side effects^(^ ^27^ ^–^ ^29^ ^)^. In addition, the increasing demand of non-fish source of *n*-3 PUFA is worth considering, in view of the beneficial effects of these FA in GDM women^(^ ^24^ ^)^, and during pregnancy in general, not to mention the worldwide increase in the number of vegetarians and vegans^(^ ^30^ ^)^.

The knowledge of how (i.e. molecular mechanisms) and where (i.e. targets) a given biocompound acts is of crucial importance to better understand the mechanisms governing the dietary impact on the metabolic system in GDM.

We found a good number of articles in English published up to August 2015 by searching in PubMed, using the key words ‘gestational diabetes mellitus’, ‘diabetes’, ‘insulin resistance’, ‘hyperglycaemia’, ‘adipokines’, ‘inflammation’, ‘microRNAs’, ‘PUFAs’, molecular mechanisms’; these key words were searched in combination with the key words ‘Mediterranean diet’, vegetables food’, ‘polyphenols’, ‘phytochemicals’, ‘bioactive compounds’. Data regarding the association between plant-derived compounds and GDM are scarce. This review discusses the current knowledge and issue about the impact of dietary polyphenols on the mechanisms and/or factors regulating glucose homoeostasis, inflammation and adipose tissue (AT) function in metabolic alterations associated with GDM. The role of *n*-3 FA in pregnancy is also addressed. From all these data, MedDiet bioactive compounds appear to be more and more useful players to be included in future research approaches designed to prevent and treat GDM.

## Dietary polyphenols

**Fig. 1:**
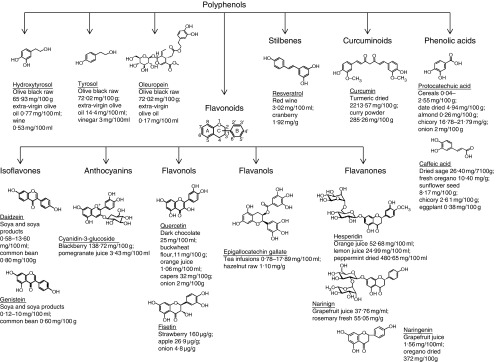
Chemical structure and main dietary source of polyphenols discussed in this review regarding their capability to modulate glucose metabolism signalling.

The MedDiet is characterised by a high intake of vegetable food^(^ ^31^ ^)^, and polyphenols are the biggest class of plant-derived bioactive compounds^(^ ^32^ ^–^ ^34^ ^)^. These phytochemicals are found in quite variable quantities in fruit, vegetables, cereals, nuts, tea, wine, chocolate, olives, extra-virgin olive oil and plant-derived foodstuffs, as well as spices and algae^(^ ^34^ ^–^ ^36^ ^)^. Even more data link polyphenol intake with both health promotion and the prevention of non-communicable diseases such as CVD, stroke, T2D and some cancers^(^ ^27^ ^–^ ^29^ ^,^ ^37^ ^)^. Polyphenols are a complex class of compounds having a phenolic ring in their structure; they can be classified on the basis of the numbers of phenol rings they contain and the structural elements that bind these rings^(^ ^32^ ^,^ ^35^ ^)^. The majority of polyphenols exist as glycosides, esters, polymers and in hydroxylated form^(^ ^35^ ^,^ ^37^ ^)^. They are extensively modified throughout stomach, small intestine, colon and liver^(^ ^38^ ^)^; thus, a single polyphenol can generate several active metabolites^(^ ^39^ ^)^. Because of their chemical structures, dietary polyphenols exert multiple activities by interacting with several molecular pathways and cellular components including microRNA (miRNA)^(^ ^28^ ^,^ ^37^ ^,^ ^40^ ^,^ ^41^ ^)^. The main classes of polyphenols are flavonoids, phenolic acids, stilbenes and lignans^(^ ^32^ ^,^ ^35^ ^)^. Flavonoids are the most abundant class of polyphenols in our diet and include different subclasses, that is, flavonols, flavones, flavanols, flavanones, anthocyanidins and isoflavones^(^ ^42^ ^,^ ^43^ ^)^ (Fig. 1). The evaluation of the actual contribution of dietary polyphenols to human health is challenging, as several factors come into play: food-related factors (i.e. amount consumed, food content and bioavailability) and host-related factors (i.e. genetics, obesity, pregnancy and gut microbiota)^(^ ^44^ ^)^. Moreover, chemical structure, synergistic effects among different polyphenols and interaction with cellular components (e.g. membrane, proteins, enzymes, receptors and transcription factors) render the evaluation quite hard^(^ ^44^ ^,^ ^45^ ^)^. An accurate measurement of the exposure to specific compounds will make it possible to associate these factors with health status and disease outcomes. To this end, efforts have been made to assess the following: (i) the level of adherence to the MedDiet, by the Mediterranean Dietary Serving Score, which accounts for the consumption of foods and food groups per meal, day or week^(^ ^46^ ^)^; (ii) the content of polyphenols in food and their metabolism^(^ ^34^ ^)^, as well as the human intake of total and specific polyphenols, by the creation of online database (e.g. Phenol-Explorer and United States Department of Agriculture database)^(^ ^34^ ^,^ ^42^ ^,^ ^43^ ^,^ ^47^ ^–^ ^49^ ^)^; and (iii) novel biomarkers for polyphenol ingestion in biological fluids, by measuring food metabolome^(^ ^50^ ^–^ ^52^ ^)^.

## Glucose homoeostasis regulation

GDM is defined as carbohydrate intolerance during pregnancy^(^ ^1^ ^)^. Pregnancy is characterised by a complex process of endocrine-metabolic changes, including physiological insulin resistance (IR), necessary to ensure the supply of nutrients to the fetus and to adequately prepare the maternal organism for childbirth and lactation^(^ ^53^ ^)^. GDM develops when the pregnant woman is not able to produce an adequate insulin response to compensate for physiological IR^(^ ^54^ ^)^. IR during pregnancy is uniquely associated with a decrease in insulin receptor substrate 1 (IRS-1) tyrosine phosphorylation, primarily because of a decreased expression of IRS-1 protein^(^ ^55^ ^)^. Nevertheless, in GDM subjects, there is an additional decrease in tyrosine phosphorylation of the insulin receptor *β* subunit, associated with further decreases in glucose transport activity^(^ ^55^ ^,^ ^56^ ^)^. Lower levels of tyrosine kinase receptor activity (30–40 %), *IRS-1* expression and activation, and *GLUT4*, in fact, have been detected in pregnancy with respect to pre-pregnancy status^(^ ^55^ ^,^ ^56^ ^)^. Specifically, IRS-1 tyrosine phosphorylation has been found to be significantly reduced in muscle from pregnant control and obese GDM subjects, as reflected by a 23 and 44 % reduction, respectively, compared with non-pregnant women^(^ ^57^ ^)^. AT, through a large arrays of secreted factors, participates in the induction and regulation of IR both in non-pregnant and pregnant subjects^(^ ^58^ ^)^. Maternal pre-pregnancy obesity decreases insulin sensitivity and positively associates with the risk of GDM^(^ ^59^ ^)^. A recent study has shown that AT inflammasome (caspase-1 and IL-1*β*) is involved in the development of IR in GDM-complicated pregnancies^(^ ^60^ ^)^. Increased IL-1*β* expression has been observed in AT from GDM women; incubation of AT with IL-1*β* results in a significant attenuation of phosphorylated IRS-1 protein expression, *GLUT4* mRNA and protein expression and glucose uptake, indicating the importance of inflammasome in the pathophysiology of GDM^(^ ^60^ ^)^.

Over the past years, the role of miRNA in gene regulation has been gaining attention; miRNA are implicated in regulating cholesterol biosynthesis, carbohydrate and lipid metabolism^(^ ^61^ ^)^, as well as in insulin production, secretion and action^(^ ^62^ ^)^ (i.e. in glucose homoeostasis)^(^ ^62^ ^–^ ^65^ ^)^, by interacting with specific mRNA targets^(^ ^66^ ^)^. Accordingly, miRNA have been explored in GDM with the aim of using them as early biomarkers of disease^(^ ^67^ ^–^ ^70^ ^)^, as they are present in the blood or other biological fluids^(^ ^67^ ^,^ ^71^ ^)^. To date, few investigations have been performed; two studies conducted in China have found decreased levels of miR-29a, -132 -and -222^(^ ^69^ ^)^, and increased levels of miR-16-5p, miR-17-5p, miR-19a-3p, miR-19b-3p and miR-20a-5p, in gestational weeks 16–19 in the serum of pregnant women who later developed GDM in gestational weeks 25–28, with respect to women who did not develop GDM^(^ ^70^ ^)^. However, hyperglycemic non-pregnant rodents have shown opposite expression of mir-29 and mir-132 families. MiR-29 family is involved in the insulin-signalling pathway; muscle, fat and liver from hyperglycaemic Goto–Kakizaki rats exhibit up-regulation of miR-29a and miR-29b^(^ ^72^ ^)^. High glucose induces the up-regulation of miR29a associated^(^ ^72^ ^)^ with the decrease in IRS-1, downstream kinase Akt and glycogen synthase kinase 3β, (GSK3β) proteins, in both rat myocytes^(^ ^73^ ^)^ and human pancreatic *β*-cells^(^ ^74^ ^)^. MiR-132 expression is up-regulated in pancreatic islets of pre-diabetic (6 weeks old) and diabetic db/db mice (14–20 weeks)^(^ ^75^ ^)^; this increase improved glucose-stimulated insulin release and increased cell proliferation, which suggests that the modification of miR-132 levels might contribute to compensatory *β*-cell mass expansion elicited in response to IR^(^ ^75^ ^)^. In GDM women, the up-regulation of miR-222 has been found to be associated with reduced protein levels of both oestrogen receptor (ER)-*α* and GLUT4 in omental AT, obtained at the time of caesarean delivery, and with increased serum estradiol levels^(^ ^76^ ^)^. In consideration of these findings, we may assume that miRNA are expressed in a species-, tissue-specific and time-dependent manner.

Furthermore, miRNA profiles of peripheral blood mononuclear cells isolated from Brazilian GDM women, obtained by using microarray platforms, have identified ten miRNA that seemed to be specific for GDM, namely, miR-101, miR-1180, miR-1268, miR-181a, miR-181d, miR-26a, miR-29a, miR-29c, miR-30b and miR-595^(^ ^67^ ^)^.The target genes of most of these miRNA are involved in insulin signalling, angiogenesis, IR and AT dysfunction^(^ ^66^ ^)^. It is noteworthy that miR-181a is increased in the serum of diabetic patients, as well as in IR cultured hepatocytes and liver^(^ ^77^ ^)^. Inhibition of miR-181a by antisense oligonucleotides restores insulin sensitivity in hepatocytes, thus providing evidence of a potential therapeutic strategy for treating IR and T2D^(^ ^77^ ^)^. On the other hand, miR-30 belonging to the miR-30 family that has a key role in angiogenesis^(^ ^78^ ^)^ might be involved in the hyper capillarisation of the placenta in women with mild hyperglycaemia^(^ ^79^ ^,^ ^80^ ^)^. Furthermore, miR-30 family members increase in abundance during the differentiation of pancreatic islet-derived mesenchymal cells into hormone-producing islet-like cell aggregates, thus indicating their participation in the regulatory signalling of the embryonic development of human pancreatic islets^(^ ^81^ ^)^. However, the miRNA modifications so far observed in GDM women result from a limited number of screened subjects (from six to twenty). In addition, people from different countries appear to have different miRNA profiles, except for miR-29, in the same metabolic condition. In conclusion, although the above data provide evidence that miRNA are promising, non-invasive biomarkers of GDM, further research with larger cohorts is warranted.

### Polyphenols effects

**Fig. 2:**
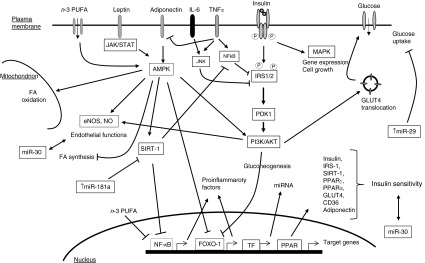
Crosstalk among signalling pathways in regulating glucose metabolism. All of the factors that appear in this scheme are potential points of action of polyphenols. 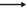, Activation; 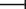, inhibition; 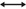, modulation; JAK/STAT, Janus kinase/signal transducer and activator of transcription; AMPK, AMP-activated protein kinase; JNK, c-Jun *N*-terminal kinase; IRS1/2, insulin receptor substrate 1/2; MAPK, mitogen-activated protein kinases; PDK1, 3-phosphoinositide-dependent protein kinase-1; PI3K, phosphatidylinositol-3-kinase; Akt, protein kinase B; SIRT-1, sirtuin 1; NO, nitric oxide; eNOS, endothelial NO synthase; FOXO1, forkhead box protein O1; TF, transcription factors; miRNA, microRNA; FA, fatty acids.

The maintenance of glucose homoeostasis is extremely important for human physiology^(^ ^82^ ^)^. Growing evidence indicates that polyphenols, contained in fruits and vegetables, might influence glucose homoeostasis by several mechanisms such as by (i) inhibiting carbohydrate digestion and glucose absorption in the intestine, (ii) stimulating insulin secretion from pancreatic *β*-cells, (iii) modulating glucose release from liver and (iv) activating insulin receptors and glucose uptake in insulin-sensitive tissues^(^ ^83^ ^)^. The bioactive food components can modify gene expression and regulate different signalling pathways, thus affecting muscle, liver, pancreatic *β*-cells, hypothalamus and AT functions, thereby regulating glucose homoeostasis^(^ ^84^ ^,^ ^85^ ^)^. This regulatory activity occurs also by modulating miRNA gene expression^(^ ^40^ ^,^ ^86^ ^–^ ^89^ ^)^ (Fig. 2). Isoflavones, anthocyanins, flavanols and catechins appear to enhance *β*-cell function and glucose tolerance in animal models and humans and to protect against diabetes^(^ ^29^ ^)^. Genistein and daidzein are the major dietary isoflavones present primarily in soya foods^(^ ^90^ ^)^. Long-term genistein exposure (1–10 µm for 48 h) improved glucose-stimulated insulin secretion (GSIS) in mouse and human pancreatic islets^(^ ^91^ ^)^; this effect appears to be mediated by cyclic AMP/protein kinase A signalling activation and increased intracellular Ca^2+^ levels^(^ ^91^ ^)^ (Table 1).

**Table 1:** Effects of dietary polyphenols on molecular mechanisms associated with gestational diabetes mellitus

Dietary compounds	Function/mechanism	Experimental model	References
Genistein (0·2 g/kg diet) or daidzein (0·2 g/kg diet) for 9 weeks	↓Blood glucose; preserve *β*-cells	NOD mice	^(^ ^105^ ^)^
Genistein (40 µm), daidzein (40 µm) or equol (40 µm) for 2 h	↓Leptin secretion	Mouse 3T3-L1 adipocytes	^(^ ^151^ ^)^
Genistein (60·8 mg)+of daidzein (16 mg)+glycitein (3·2 mg) of for 6 months	↓Leptin; ↓TNF*α*; ↑adiponectin	Healthy obese postmenopausal women	^(^ ^156^ ^)^
Genistein (50 µm), daidzein (50 µm) or glycitein (50 µm) pretreatment for 1 h	Inhibit TNF*α*-mediated adiponectin reduction: genistein via JNK, daidzein via Foxo1	TNF*α*-treated mouse 3T3-L1 adipocytes	^(^ ^152^ ^)^
Genistein (1–10 µm) for 48 h	↑GSIS, cAMP/PKA activation	Mouse and human pancreatic islets	^(^ ^91^ ^)^
EGCG (10 µm) for 48 h	Activates IRS-2; ↑GSIS; activates AMPK	Hyperglycaemia in rat *β*-cells line RIN-m5F	^(^ ^92^ ^)^
EGCG (20–40 µm) for 24 h	↑Glucose uptake; ↑GLUT4; reverts IRS-1 inhibition; AMPK activation	Rat muscle cells L6-dexamethasone-treated	^(^ ^94^ ^)^
EGCG (50 mg/l) for 5 h	↓mir-30b	Human hepatocarcinoma cells HepG2	^(^ ^78^ ^)^
EGCG (10 µm), cocoa (10 µm) or grape seed (10 µm) proanthocyanidin, for 24 h	↓mir-181d and mir-222	HepG2	^(^ ^95^ ^)^
C3G (2 g/kg diet) for 8 weeks	↑Serum adiponectin ↑Endothelial function	db/db mice	^(^ ^146^ ^)^
C3G (50 µm) for 24 h	↑Adiponectin by activating Sirt-1 and Foxo1	Mouse 3T3-L1 adipocytes	^(^ ^146^ ^)^
C3G (150 μg/10 g) of body weight twice per day, for 30 d	↓Blood glucose	STZ-diabetic mice	^(^ ^97^ ^)^
C3G (0·5 µm) pretreatment for 12 h	↑PDX1; ↑insulin-like growth factor-II; ↑insulin	Rat pancreatic INS-1 cells	^(^ ^97^ ^)^
C3G (10, 50 µm) or PCA (100 µm), for 18 h	↑GLUT4; ↑PPAR*γ* activity; ↑adiponectin; glucose uptake	Mouse 3T3-L1 adipocytes, human adipocytes	^(^ ^98^ ^,^ ^100^ ^)^
Proanthocyanidin (300 mg/d) for 2 weeks	↑mir-181a; ↓mir-29a	apoE–/– mice (in liver)	^(^ ^88^ ^)^
Anthocyanins (320 mg/d) for 12 weeks	↑Serum adiponectin	T2D patients	^(^ ^146^ ^)^
Whole blueberry (45 g/d=668 mg anthocyanins) for 6 weeks	↓Blood glucose; ↑insulin sensitivity	Obese, insulin-resistant subjects	^(^ ^107^ ^)^
Grape seed (100 mg/l) or cocoa (100 mg/l) proanthocyanidin extract , for 5 h	↓mir-30b	HepG2	^(^ ^78^ ^)^
Fisetin (1 nm–10 µm) for 24 h	↑Adiponectin secretion; ↑PPAR activation; Sirt-1 activation	Mouse 3T3-L1 adipocytes	^(^ ^147^ ^)^
Caffeic acid (10^−6^ m) for 72 h	↑GSIS; ↑Akt2; ↑INS-1 mRNA expression	Hyperglycaemia in INS-1 cells	^(^ ^101^ ^)^
Caffeic acid (300 mg/d), 2 weeks supplementation	↓mir-222; ↓mir-29a; ↓mir-30b	apoE–/– mice (in liver)	^(^ ^88^ ^)^
Naringenin (10^−6^ m)	↑GSIS; ↑Glut-2; ↑Akt1; ↑GK; ↑Kt1; mRNA expression	Hyperglycaemia in rat pancreatic INS-1 cells	^(^ ^101^ ^)^
Naringin (80 µm pretreatment) for 2 h	↓Leptin and leptin receptor and p38-MAPK activation	High glucose in rat H9c2 cardiac cells	^(^ ^153^ ^)^
Naringin (30 mg/d) for 2 weeks	↑ mir-181a; ↓mir-132; ↓mir-29a; ↓mir-30b	apoE–/– mice (in liver)	^(^ ^88^ ^)^
Naringin (50 mg/kg) or hesperidin (50 mg/kg) for 4 weeks	↓Blood HbA1c %; ↓serum TNF*α* and IL-6	Diabetic rats	^(^ ^154^ ^)^
Hesperidin (30 mg/d) for 2 weeks	↓mir-181a; ↓mir-222; ↓mir-222 ↓mir-30b	apoE–/– mice (in liver)	^(^ ^88^ ^)^
Quercetin (10^−6^ m) for 72 h	↑GSIS, SGlut-2, ↑SGlut-2,2 ↑INS-1 mRNA expression	Hyperglycaemia in INS-1 cells	^(^ ^101^ ^)^
Quercetin (30 mg/d) for 2 weeks	↑mir-181a; ↓mir-29a; ↓mir-30b	apoE–/– mice (in liver)	^(^ ^88^ ^)^
Curcumin (5–15 µm) for 24 h	↑GSIS; Glut-2; GIRS1; ↑PI3K and Akt activation	High glucose in INS-1 cells	^(^ ^102^ ^)^
Curcumin (30 mg/d) for 2 weeks	↓mir-181a, ↓ mir-29a, ↓mir-30b	apoE–/– mice (in liver)	^(^ ^88^ ^)^
Curcumin (15 µm for 48 h)	↑mir-26a	Human lung adenocarcinoma cell line A549	^(^ ^103^ ^)^
Curcumin (10 µm) for 72 h	↑mir-181a	Human pancreatic cancer cells BxPC-3	^(^ ^104^ ^)^
Resveratrol (50 µm) for 24 h	↑Adiponectin via Akt/FOXO1 and the AMPK signalling pathways	3T3-L1 adipocytes	^(^ ^142^ ^)^
Resveratrol (0·1–10 µm), 1 h pretreatment	↓TNF*α* and IL-6 ↓NF-*κ*B and ERK1/2 activation	3T3-L1 adipocytes exposed to conditioned medium (i.e. from LPS-treated RAW264·7 macrophages)	^(^ ^144^ ^)^
Resveratrol (0·1–1 µm) for 24 h	Prevents hyperpermeability; ↑NO production; ↑eNOS activity	High-glucose-treated BAEC	^(^ ^145^ ^)^
36 % fat supplemented with 0·37 % resveratrol	↑Placental DHA uptake; ↑HA u activity; ↑mRNA expression of DHA receptors	High-fat diet (36 % fat supplementated with 0·37 % resveratrol) in pregnant non-human primates	^(^ ^193^ ^)^
Tyr (0·1–10 µm) for 1 h pretreatment	↑Adiponectin; ↑PPAR*γ* activity; ↓JNK activation	Human adipocytes TNF*α* treated	^(^ ^149^ ^)^
Tyr, or OL, or HT, or Tax (50 µm each) for 24 h	↓Proliferation and migration; HT and Tax↓VEGFR2, ERK1/2 and JNK phosphorylation	VEGF-treated HUVEC	^(^ ^150^ ^)^

↑, Increases; ↓, decreases; NOD, non-obese diabetic; JNK, c-Jun *N*-terminal kinase; Foxo1, forkhead box protein O1; GSIS, glucose-stimulated insulin secretion; cAMP, cyclic AMP; PKA, protein kinase A; EGCG, epigallocatechin-3-gallate; IRS1/2, insulin receptor substrate 1/2; AMPK, AMP-activated protein kinase; C3G, cyanidin-3-glucoside; Sirt-1, sirtuin 1; STZ, streptozotocin; PDX1, pancreatic and duodenal homeobox-1; PCA, protocatechuic acid; T2D, type 2 diabetes; Akt, protein kinase B; INS-1, insulin 1; GK, glycerol kinase; p38 MAPK, p38 mitogen-activated protein kinase; HbA1c, glycated Hb; PI3K, phosphatidylinositol-3-kinase; ERK1/2, extracellular signal-regulated kinase; LPS, lipopolysaccharides; NO, nitric oxide; eNOS, endothelial NO synthase; BAEC, bovine artery endothelial cells; Tyr, tyrosol; OL, oleuropein; HT, hydroxytyrosol; Tax, taxifolin; VEGFR2, vascular endothelial growth factor receptor 2; VEGF, vascular endothelial growth factor; HUVEC, human umbilical vein endothelial cells.

The flavanol epigallocatechin gallate (EGCG), a main component of green tea extract, preserves the insulin secretory machinery; EGCG (10 µm for 48 h) stimulates the activation of the IRS-2 signalling in rat insulinoma pancreatic *β*-cells (RIN-m5F), also under chronic hyperglycaemia^(^ ^92^ ^)^. EGCG increases the activity of AMP-activated protein kinase (AMPK) through Thr^172^ phosphorylation, further strengthening the hypothesis that AMPK is involved in counteracting the glucolipotoxicity induced by high-glucose condition^(^ ^92^ ^)^. AMPK is an energy-sensing enzyme recognised as a master regulator of whole-body energy homoeostasis; its activation increases GLUT4 expression and membrane translocation in skeletal muscle, thereby improving glucose uptake^(^ ^93^ ^)^. In this regard, AMPK has been considered as one of the targets of the insulin-sensitising drugs metformin and thiazolidinediones^(^ ^93^ ^)^.

EGCG (20, 40 μm for 24 h) improved the insulin-stimulated glucose uptake also in dexamethasone-treated skeletal muscle cells L6, associated with an increased AMPK phosphorylation and GLUT4 membrane translocation^(^ ^94^ ^)^. Furthermore, the dexamethasone-stimulated inactivation of IRS-1 because of increased Ser^307^ phosphorylation has been significantly reversed by EGCG treatment^(^ ^94^ ^)^. Results from the miRNA profiling suggest that EGCG may exert its biological functions through up- or down-regulating multiple miRNA as well^(^ ^95^ ^)^. In particular, EGCG (50 mg/l for 5 h), grape seed (100 mg/l for 5 h) and cocoa proanthocyanidin extracts (100 mg/l for 5 h) down-regulated miR-30b expression in human hepatocellular carcinoma HepG2 cells^(^ ^78^ ^)^. A longer EGCG (100 μm for 24 h) treatment reduced miR-181d and miR-222 expression as well, in HepG2 cells^(^ ^95^ ^)^.

Anthocyanins are widely distributed in the human diet through berries, fruits, vegetables and red wine^(^ ^96^ ^)^. Pretreatment with bayberry fruit extracts (0·5 μmol/l cyanidin-3-glucoside (C3G) for 12 h) up-regulated pancreatic duodenal homeobox-1 gene expression, which is associated with increased levels of insulin-like growth factor-II gene transcript and insulin, in rat insulinoma cell line INS-1*β*-cells^(^ ^97^ ^)^.

We have shown that C3G (10·50 µm for 18 h) and its metabolite protocatechuic acid (PCA; 100 µm for 18 h) increased glucose uptake and GLUT4 membrane translocation in murine adipocytes and visceral human adipocytes^(^ ^98^ ^–^ ^100^ ^)^. Specifically, PCA appeared to exert insulin-like effects by stimulating IRS-1 tyrosine phosphorylation and Akt activation^(^ ^98^ ^–^ ^100^ ^)^.

Caffeic acid, naringenin and quercetin (10^−6^ µm for 72 h each), present in many plants, enhance GSIS and glucose sensitivity in rat pancreatic INS-1E cells^(^ ^101^ ^)^. These compounds differently modulate gene expression profiles to improve *β*-cell survival and function during glucotoxicity (e.g. naringenin and quercetin up-regulated GLUT2, glucokinase, AKT1, whereas caffeic acid increased AKT2 and all the three compounds increased INS-1, mRNA expression)^(^ ^101^ ^)^. Similarly, curcumin (5–15 µm for 24 h), a yellow pigment isolated from *Curcuma Longa*, increases insulin gene expression and GSIS in a dose-dependent manner in INS-1 cells^(^ ^102^ ^)^. In doing so, curcumin up-regulates the expression of GLUT2, the phosphorylation of IRS-1, phosphatidylinositol-3-kinase (PI3K) and Akt, suggesting that curcumin prevents the high-glucose-induced reduction of insulin expression and secretion by activating the PI3K/Akt/GLUT2 pathway in INS-1 cells^(^ ^102^ ^)^. Curcumin treatment modulates the expression profile of several miRNA, including up-regulation of miRNA-26a in A549 human lung adenocarcinoma cell line (curcumin: 15 µm for 48 h)^(^ ^103^ ^)^ and miR-181a in BxPC-3 human pancreatic carcinoma cell line (curcumin: 10 µm for 72 h)^(^ ^104^ ^)^.

Consistent with *in vitro* studies, animal intervention studies showed that supplementation with genistein (0·2 g/kg diet) or daidzein (0·2 g/kg diet) for 9 weeks preserved *β*-cells and reduced blood glucose levels by inducing insulin secretion, in non-obese diabetic mice^(^ ^105^ ^)^. Similarly, administration of 0·1 ml of bayberry fruit extracts (containing 150 μg of C3G)/10 g of body weight twice per day for 30 d significantly reduced blood glucose and improved glucose tolerance in streptozotocin-induced diabetic mice^(^ ^97^ ^)^.

Changes in the expression of a large number of different miRNA have been observed after each polyphenol supplementation^(^ ^88^ ^)^. MiRNA profile expression was evaluated in the livers of wild-type (C57B6/J) mice or apoE–/– mice fed diets supplemented with different polyphenols (including quercetin, hesperidin, naringenin, proanthocyanidin, caffeic acid, curcumin)^(^ ^88^ ^,^ ^106^ ^)^. MiR-29a and miR-30b expression were down-regulated by almost all the tested polyphenols; miR-222 was down-regulated by caffeic acid (300 mg/d for 2 weeks) and hesperidin (30 mg/d for 2 weeks); miR-181a was down-regulated by curcumin (30 mg/d for 2 weeks) and hesperidin and up-regulated by naringin (30 mg/d for 2 weeks), quercetin (30 mg/d for 2 weeks) and proanthocyanidin (300 mg/d for 2 weeks); miR-132 was up-regulated by naringin^(^ ^88^ ^)^. This study highlights the importance of miRNA as modulators of dietary polyphenols activities, *in vivo* ^(^ ^88^ ^)^.

Human intervention trials and cohort studies have reported that dietary supplementation with 22·5 g of whole blueberry twice daily (1462 mg of total phenolics, 668 mg of anthocyanins) for 6 weeks resulted in blood glucose concentration reduction and in insulin sensitivity improvement in obese, non-diabetic, IR subjects^(^ ^107^ ^)^. Data from three prospective cohort studies involving 200 000 US men and women showed that a higher consumption of anthocyanin-rich fruit is associated with a lower risk of T2D^(^ ^108^ ^)^. Collectively, anthocyanins and its glycosides, alone or in combination, improve glucose homoeostasis by influencing *β*-cell mass and function, insulin sensitivity, glucose uptake and insulin signalling^(^ ^85^ ^)^.

There is no doubt that dietary polyphenols have beneficial effects on *β*-cell function and glucose homoeostasis taking into account that most of them are capable of modulating almost all the players, including several miRNA, in the glucose uptake machinery. Although promising data have been obtained so far, no information on the effects of these bioactive compounds in GDM setting are available as yet.

## Adipokines and gestational diabetes

It is well known that adipocyte-derived cytokines (adipokines) are involved in the modulation of a wide range of physiological processes including lipid metabolism, atherosclerosis, angiogenesis and insulin sensitivity^(^ ^109^ ^,^ ^110^ ^)^. The human gestational tissues, as well as AT, produce and release a large array of pro-inflammatory cytokines; the activation of some inflammatory pathways is necessary to induce maternal IR, which is required for the progression of normal gestation^(^ ^9^ ^,^ ^111^ ^,^ ^112^ ^)^. GDM associates with the amplification of the low-grade inflammation already existing in normal pregnancy^(^ ^113^ ^,^ ^114^ ^)^. Indeed, serum TNF*α*, IL-1*β* and IL-6 levels are higher in GDM women as compared with control pregnant women^(^ ^115^ ^)^. Notably, pre-pregnant obesity in GMD women worsens this inflammatory picture by up-regulating TNF*α*, IL-1*β* and/or leptin, and by down-regulating genes involved in AT lipid metabolism such as *PPARα*, *PPARδ*, *PPARγ*, retinoid X receptor-*α* (*RXRα*) and sterol regulatory element-binding protein-1c (*SREBP1c*), in AT^(^ ^109^ ^)^.

Over the past years, various adipokines such as adiponectin and leptin have been shown to play a role in normal pregnancy, as well as in complications of pregnancy including GDM^(^ ^9^ ^,^ ^110^ ^,^ ^116^ ^,^ ^117^ ^)^. Low adiponectin serum levels appear to be linked with T2D and IR^(^ ^118^ ^)^. A study conducted on 445 pregnant women demonstrated that lower levels of adiponectin during the first trimester of pregnancy are associated with increased risk of developing GDM during the second trimester, independently of other early pregnancy major risk factors^(^ ^119^ ^)^. Further, a nested case–control study showed that lower adiponectin levels measured 6 years before pregnancy were associated with a 5-fold increased risk of developing GDM^(^ ^120^ ^)^. Thus, an early low adiponectin expression, which most likely reflects pre-existing IR, could be considered a useful biomarker to identify women at high risk for GDM and allow for early therapeutic interventions^(^ ^120^ ^)^. Adiponectin acts as an insulin-sensitising agent, and its action is complex and incompletely defined, but the collected evidence suggests that, by binding with its receptors, adiponectin activates three key signalling pathways in muscle and liver: AMPK, p38 mitogen-activated protein kinase (p38 MAPK) and PPAR^(^ ^121^ ^)^. The activation of these pathways results in FA oxidation (FAO) and glucose uptake in skeletal muscle, and in the inhibition of gluconeogenesis in the liver^(^ ^121^ ^)^. Reduced adiponectin could be held accountable for the lower FAO observed in placentas of GDM women compared with the control group^(^ ^122^ ^)^. Adiponectin gene expression, processing and secretion is up-regulated by PPAR*γ* agonist; PPAR*γ* activation results in increased insulin sensitivity, in skeletal muscle and liver, by inducing the release of insulin-sensitising adipokines including adiponectin^(^ ^123^ ^)^. Administration of adiponectin to diabetic mice^(^ ^124^ ^)^ and obese pregnant mice enhances insulin activity^(^ ^125^ ^)^, prevents fetal overgrowth^(^ ^125^ ^)^, normalises placental insulin/mammalian target of rapamycin complex 1 (mTORC1), PPAR*α* signalling and placental nutrient transport^(^ ^125^ ^)^. Adiponectin also mediates the crosstalk between AT and the vessel wall; thus, in virtue of its pleiotropic activities on multiple targets, it seems to have a protective role against vascular dysfunction induced by obesity and diabetes^(^ ^126^ ^)^.

TNF*α* and leptin have been suggested as the strongest predictors of pregnancy-related IR^(^ ^127^ ^,^ ^128^ ^)^. Hyperleptinaemia in early pregnancy (13 weeks) appears to be a good predictor of GDM risk, independent of maternal adiposity^(^ ^129^ ^,^ ^130^ ^)^. In week 30 of pregnancy, leptin levels have been found to be similar in both GDM and no-GDM subjects, but they remained elevated after delivery in the GDM group^(^ ^131^ ^)^.

Recent systematic reviews have highlighted higher and lower levels of plasma leptin and adiponectin, respectively, in the first or second trimester of pregnancy^(^ ^132^ ^)^, and this condition persisted, associated also with high TNF*-α* levels, in the late second or third trimester of pregnancy^(^ ^133^ ^)^, in GDM patients compared with normal pregnancies. These findings corroborate the role exerted by the altered balance pro-inflammatory/anti-inflammatory factors in the impaired glucose homoeostasis in GDM.

Increased levels of leptin have been found in visceral AT of obese and GDM women^(^ ^109^ ^)^. Leptin, predominantly produced by adipocytes, is considered an essential factor in maintaining energy homoeostasis, as it regulates food intake and energy expenditure via specific receptors in the hypothalamus^(^ ^134^ ^)^. Leptin acts by activating Janus kinase/signal transducer and activator of transcription signalling, although other important intracellular signalling including PI3K, AMPK and MAPK appear to be involved as well^(^ ^134^ ^)^.

In GDM women, TNF*α* and leptin levels are elevated; the increased TNF*α* expression causes a chronic inflammatory environment with enhanced leptin production, which, in turn, increases TNF*α* and IL-6 production by monocytes^(^ ^135^ ^)^, generating a vicious circle that amplifies the inflammatory situation and worsens the metabolic dysfunction in GDM. Although the significance of leptin and adiponectin is relatively well characterised as for their effects on glucose and lipid metabolism in diabetes, the molecular mechanisms by which these adipokines exert their effects on insulin action are not completely defined. Much more research is needed especially in the light of the growing list of molecules identified as adipokines, for example, visfatin, apelin, retinol-binding protein 4, vaspin, omentin and adiposity FA-binding protein 4 (FABP4), which renders the comprehension of the existing network and the interactions among them more complicated ^(^ ^9^ ^,^ ^136^ ^,^ ^137^ ^)^.

### Polyphenols effects

The beneficial effects of MedDiet dietary compounds on diabetes are associated with reduced biomarkers of inflammation^(^ ^138^ ^–^ ^140^ ^)^.

Taking into account the importance of the insulin-sensitising effects of adiponectin, enhancing adiponectin/AdipoR function may be an interesting therapeutic strategy against IR^(^ ^141^ ^)^. Dietary compounds such as fish oil, linoleic acid (LA), seed extract, green tea extract and resveratrol (RSV) elevated adiponectin concentration; specifically, RSV (50 µm for 24 h) treatment stimulated adiponectin expression and reduced mRNA levels of leptin in 3T3-L1 adipocytes^(^ ^142^ ^,^ ^143^ ^)^. The stimulatory effect of RSV has been shown to be mediated via histone deacetylase sirtuin 1 (Sirt-1)-independent mechanism and is mainly because of phosphoinositide-dependent protein kinase-1/Akt signalling pathway suppression, in the activation of the transcription factor forkhead box O-1 (FOXO1), and the AMPK signalling pathway^(^ ^142^ ^)^. RSV (0·1–10 μm) reduced the expression and release of IL-6 and TNF*α* in RAW 264.7 macrophages and 3T3-L1 cells, in association with a reduced extracellular signal-regulated protein kinases 1/2 (ERK1/2) and NF-*κ*B activation^(^ ^144^ ^)^. The high-glucose-induced nitric oxide (NO) reduction was reverted by RSV (0·1–10 μm for 24 h) incubation in bovine artery endothelial cells, because of enhanced endothelial NO synthase mRNA and activity^(^ ^145^ ^)^. Altogether, these findings strongly indicate that RSV could be a therapeutic supplement to counteract the diabetic condition.

C3G treatment (12·5, 25, 50 µm for 24 h) induced adiponectin expression in 3T3-L1 and human adipocytes^(^ ^98^ ^,^ ^146^ ^)^. As regards the molecular mechanisms, C3G induced FOXO1 deacetylation and transcriptional activity, as well as increased PPAR*γ* activity^(^ ^98^ ^)^, which, in turn, brought about adiponectin expression and secretion, in adipocytes^(^ ^146^ ^)^. In addition, flavonoid fisetin (0·1–10 μm for 24 h) increased adiponectin gene transcription by inducing SIRT-1-deacetylase activity and PPAR activation, in mouse adipocytes^(^ ^147^ ^,^ ^148^ ^)^.

Virgin olive oil components such as hydroxytyrosol (HT; 0·1–20 μm for 1 h pretreatment) and oleic acid (100 μm for 48 h pretreatment) alone or in combination in human adipocytes can prevent TNF*α*-induced down-regulation of adiponectin by attenuating PPAR*γ* suppression mediated by c-Jun *N*-terminal kinase (JNK)^(^ ^149^ ^)^. Moreover, HT, oleuropein, taxifolin and tyrosol (50 µm pretreatment for 24 h each) showed anti-angiogenesis activities as well^(^ ^150^ ^)^. Indeed, all these compounds suppressed angiogenesis in human umbilical vein endothelial cells by inhibiting vascular endothelial growth factor receptor 2 (VEGFR-2) signalling pathway. Specifically, HT and taxifolin inhibited VEGF-dependent tyrosine phosphorylation of VEGFR-2 by impairing phosphorylation of p42/p44 MAPK (ERK1/2) and p46/p54 stress-activated protein kinase (SAPK)/JNK^(^ ^150^ ^)^. Altogether, these data provide further molecular evidence of the beneficial effects of olive oil consumption in the MedDiet in the treatment of metabolic diseases.

The soya isoflavones and their aglycones daidzein and genistein, as well as the equol metabolite (40 µm for 2 h for each), inhibited leptin secretion in mouse adipocytes^(^ ^151^ ^)^. A mechanistic study carried out in 3T3-L1 adipocytes demonstrated that genistein and daidzein inhibit TNF*α*-mediated down-regulation of adiponectin through different mechanisms; genistein inhibited TNF*α*-induced JNK signalling and daidzein inhibited TNF*α*-induced down-regulation of FOXO1^(^ ^152^ ^)^. Naringin (80 µm pretreatment for 2 h) incubation down-regulated the high-glucose-induced leptin expression and inhibited leptin-induced activation of the p38 MAPK pathway in H9c2 cardiac cells^(^ ^153^ ^)^. An *in vivo* study showed that oral administration of naringin (50 mg/kg for 4 weeks) or hesperidin (50 mg/kg for 4 weeks) in diabetic rats significantly reduced the percentage of blood glycated Hb, and serum levels of TNF*α* and IL-6^(^ ^154^ ^)^. In humans, purified anthocyanin (320 mg/d for 12 weeks) supplementation significantly increased serum adiponectin concentrations in patients with T2D^(^ ^146^ ^)^.

The metabolites of polyphenols can be useful biomarkers of dietary intake to evaluate individual response to specific compounds; for example, equol is not produced in all adults who consume soya foods^(^ ^155^ ^)^. A recent study has shown that, in pre-diabetic and diabetic female population, the ‘equol producers’ subjects exhibited lower levels of leptin as compared with non-producers, indicating an association between isoflavone intake and leptin levels^(^ ^155^ ^)^. In healthy obese postmenopausal women, physical activity, diet and daily oral intake of a soya isoflavone extract (60·8 mg of genistein, 16 mg of daidzein and 3·2 mg of glycitein) resulted in reduced levels of serum leptin and TNF*α* associated with a significant increase in adiponectin, after 6 months of treatment^(^ ^156^ ^)^.

A recent and exhaustive review describes the inhibitory effects of certain polyphenols on TNF*α*-activated inflammatory pathways both *in vitro* and *in vivo*, and in human studies^(^ ^157^ ^)^.

## The health effects of PUFA

A substantial amount of FA is required as additional source of energy to support fetal cellular growth, and gestational hyperlipidaemia normally occurring during late pregnancy enhances placental access to FA^(^ ^158^ ^,^ ^159^ ^)^. GDM associates with an altered maternal lipid profile and affects the quantity and/or quality of lipids transferred to the fetus^(^ ^160^ ^)^. In GDM, a positive correlation between maternal TAG and NEFA levels, and fetal growth and fat mass, has been found even in diabetic mothers with appropriate glycaemic control^(^ ^160^ ^)^. Fetus development depends on the maternal supply of essential PUFA such as LA (18 : 2 *n*-6), *α*-linolenic acid (ALA, 18 : 3 *n*-3) and long-chain (LC)-PUFA, EPA (20 : 5 *n*-3), DHA (22 : 6 *n*-3) and arachidonic acid (AA, 20 : 4 *n*-6)^(^ ^161^ ^)^. The placenta regulates adequate LC-PUFA delivery to the fetus in a directional, preferential and timely manner^(^ ^161^ ^)^, preferentially transferring *n*-3 LC-PUFA^(^ ^159^ ^)^. In GDM women, the placental transfer of LC-PUFA appears to be altered, with an impaired maternal-fetal transfer of DHA, which might affect neurodevelopment programming in the offspring^(^ ^162^ ^)^. A reduced concentration of LC-PUFA in maternal, placental and fetal compartments in GDM women compared with healthy pregnant women has been highlighted^(^ ^163^ ^,^ ^164^ ^)^.

However, increased, unchanged or lower hyperlipidaemia in diabetic pregnant women *v*. non-diabetic pregnant women has been observed too^(^ ^160^ ^)^. In this regard, it has been hypothesised that the degree of metabolic control and sex hormonal dysfunction may influence the different degree of dyslipidaemia observed in diabetic pregnant women^(^ ^160^ ^)^. NEFA are taken up by the placenta and FA are released from maternal lipoproteins by endothelial lipase (EL) and lipoprotein lipase (LPL), which are present in the maternal-facing microvillus membrane of the syncytiotrophoblast^(^ ^162^ ^)^. Increased EL mRNA has been found in placenta from obese women with GDM compared with lean GDM women or normoglycemic pregnant women^(^ ^165^ ^)^. In contrast, a recent study has shown that no difference in the expression of LPL and EL exists between GDM and normoglycaemic placentas, suggesting that these lipases are not involved in the increased newborn adiposity^(^ ^166^ ^)^. NEFA may enter the cell by passive diffusion or by several plasma membrane-located transport/binding proteins including FA translocase (FAT/CD36), plasma membrane FABP(pm), FA transport proteins (FATP1–6) and intracellular FABP^(^ ^167^ ^)^. DHA and EPA are endogenous ligands of several transcription factors such as PPAR, RXR and SREBP1^(^ ^168^ ^)^. A decreased expression of genes involved in FA uptake, intracellular transport, storage and synthesis (e.g. LPL, FATP2, FATP6, FABPpm, acyl-CoA synthetase long-chain family member 1), and of transcription factors involved in lipid metabolism regulation (e.g. *liver X receptor* (*LXRα*), *PPARα*, *PPARδ*, *PPARγ*, *RXRα*, *SREBP1c*), has been observed in AT obtained from obese pregnant women and women with GDM^(^ ^109^ ^)^. These results indicate that pre-existing maternal obesity and GDM are associated with abnormal AT lipid metabolism, which may play a role in the pathogenesis of both diseases^(^ ^109^ ^)^.

### Dietary effects

PUFA are fundamental for human beings, and particularly important is the balance of *n*-6:*n*-3 FA in maintaining homoeostasis, normal development and mental health throughout the lifecycle^(^ ^161^ ^)^. Different studies have led to the conclusion that humans currently need a diet containing *n*-3 and *n*-6 PUFA in a ratio of about 1:5; on the contrary, in Western diets, this ratio is 1:10–20, indicating a deficiency in *n*-3 FA and/or an excess of *n*-6 FA^(^ ^169^ ^)^. The essential FA, LA and ALA cannot be synthesised by humans and therefore must be supplied through the diet; a series of sequential desaturation and elongation reactions acting in concert transform LA and ALA into their unsaturated derivatives, namely AA from LA, EPA and DHA from ALA^(^ ^170^ ^)^. EPA and DHA are abundantly present in specific fishes, for example, fresh tuna, salmon and mackerel, as well as in fish oil and nuts^(^ ^171^ ^,^ ^172^ ^)^. Nuts, vegetables, vegetable oils, for example, soyabean, flaxseed and rapeseed oil, and cereals are also food sources of ALA and LA^(^ ^49^ ^,^ ^170^ ^)^. Walnuts are widely consumed in Mediterranean regions and are rich in ALA (2·95 g/28·4 g)^(^ ^173^ ^)^. In the PREDIMED (PREvención con DIeta MEDiterránea) trial that involved subjects at high risk for CVD, a diet rich in fats of vegetable origin, olive oil and mixed nuts (30 g/d, as 15 g walnuts, 7·5 g hazelnuts and 7·5 g almonds) has been shown to be beneficial in the management of the metabolic syndrome^(^ ^174^ ^)^. The fat content of vegetables is low, but a high consumption of vegetables will result in a substantial intake of ALA. The content of ALA in some edible plants growing in the Mediterranean regions is remarkable, as in the case with purslane (300–400 mg ALA/100 g)^(^ ^175^ ^,^ ^176^ ^)^.

Different cohort studies have shown a positive association between the adherence to MedDiet style, and plasma DHA and EPA content, indicating that the MedDiet ensures higher *n*-3 PUFA bioavailability than Western diets^(^ ^177^ ^–^ ^180^ ^)^.

The European Commission and the International Society for the Study of Fatty Acids and Lipids specifically recommend that pregnant and lactating women consume a minimum of 200 mg DHA/d^(^ ^181^ ^)^. Several researches indicate that maternal dietary supplementation with *n*-3 PUFA during pregnancy can reduce the risk of pregnancy complications by limiting placental inflammation and oxidative stress^(^ ^159^ ^,^ ^182^ ^,^ ^183^ ^)^, with positive action on insulin function, improvement of glucose tolerance and lipid profiles^(^ ^184^ ^,^ ^185^ ^)^ (Table 2).

**Table 2:** Maternal *n*-3 fatty acids (FA) supplementation

Subjects	Study	Outcomes	References
Healthy pregnant women	200 mg of DHA daily from 8 weeks of gestational age until delivery	↑00 mg of FA and DHA in maternal blood; ↓arachidonic acid levels	^(^ ^182^ ^)^
Obese women with polycystic ovary syndrome	4 g *n*-3 PUFA (720 mg EPA and 480 mg DHA)/daily for 8 weeks	↑g *n*-3 Padiponectin levels; ↓serum glucose, insulin, cholesterol and TAG	^(^ ^184^ ^)^
Obese rats	Diet containing 5 % *n*-3 PUFA daily for 16 weeks	↓Diet containing leptin, insulin and cholesterol; ↑plasma adiponectin	^(^ ^185^ ^)^
Type 2 diabetes pregnant women	600 mg of fish oil-derived DHA daily from first trimester until delivery	Prevents decline in maternal DHA during pregnancy; ameliorates red cell membranes	^(^ ^186^ ^)^
Rats	Diet containing 32·2 % *n*-2 PUFA (5·4 %EPA and 23·8 %DHA) daily from 1 d of pregnancy to 17 or 22 d of gestation	↑Resolvin and protectin in placenta	^(^ ^159^ ^)^
Pregnant women	3·7 g of fish oil-derived *n*-3 PUFA (56 % DHA and 27·7 % EPA), from 20 week of gestation until delivery	↑0·7 g fish oil-derived *n*-3 PUFA (56 % specialised pro-resolving lipid mediators’)	^(^ ^189^ ^)^
Gestational diabetes women	1 g PUFA (180 mg EPA and 120 mg DHA) from 24–28 weeks of gestation for 6 weeks	↓Serum insulin, ↓homoeostasis model of assessment–insulin resistance; ↓high-sensitivity C-reactive protein and a better newborns outcome	^(^ ^24^ ^,^ ^190^ ^)^
Transgenic mice model synthesising *n*-3 PUFA	Five streptozotocin injections	Prevents streptozotocin-induced diabetes	^(^ ^194^ ^)^

↑, Increases; ↓, decreases.

A daily dose of 600 mg of DHA, starting from the first trimester until delivery, can ameliorate red cell membrane anomaly in pregnant women with T2D and in neonates, and it prevents the decline of maternal DHA during pregnancy^(^ ^186^ ^)^. These results suggest that DHA supplementation should be started in the antenatal care of pregnant women with T2D. An Italian pilot study revealed that daily administration of 200 mg of DHA, from the 8th week until delivery, reduced AA levels in erythrocytes and plasma during gestational time^(^ ^182^ ^)^. Thus, the increased DHA levels in the maternal plasma and the consequent reduction of AA-derived eicosanoids decrease inflammation, with a beneficial effect on fetal development and growth, and IR of amniochorial membranes as well^(^ ^182^ ^)^. New identified PUFA-derived mediators (specialised pro-resolving lipid mediators), namely resolvins, protectins, maresins and lipoxins, are involved in inflammatory resolution^(^ ^187^ ^)^. It has been observed that maternal dietary supplementation with *n*-3 PUFA might help placenta in resolving inflammation by increasing the levels of these pro-resolving mediators, in rat^(^ ^188^ ^)^ and in human placenta^(^ ^189^ ^)^. GDM women who received 1000 mg/d *n*-3 FA supplementation (containing 180 mg of EPA and 120 mg of DHA) for 6 weeks, starting in weeks 24–28 of gestation, showed a significant decrease in serum insulin levels and in HOMA index (homoeostasis model of assessment–IR)^(^ ^24^ ^)^, high-sensitivity C-reactive protein (CRP) and better newborn outcome^(^ ^190^ ^)^, compared with placebo groups.

The need of higher PUFA intake in pregnancy is highlighted by data collected from 600 women participating in the Alberta Pregnancy Outcomes and Nutrition study^(^ ^191^ ^)^. Through the development of a dietary database for *n*-3 PUFA, the authors evaluated PUFA intake each trimester of pregnancy and 3 months postpartum: results indicate that the majority of women did not meet the European Union recommendation for DHA during pregnancy^(^ ^191^ ^)^.

The mechanism through which *n*-3 FA intake might influence insulin metabolism is not well known. It has been hypothesised that *n*-3 PUFA supplementation might affect insulin metabolism and lipid profiles by activating AMPK^(^ ^192^ ^)^. In agreement with this finding, a recent study showed that RSV supplementation, before, and throughout, pregnancy increased placental DHA uptake capacity in pregnant non-human primates fed a high-fat diet (i.e. 36 % fat supplemented with 0·37 % RSV)^(^ ^193^ ^)^. This effect was associated with increased AMPK activity and *FATP-4*, *CD36* and *FABPpm* mRNA expression^(^ ^193^ ^)^. In addition, *n*-3 PUFA might modulate gene expression through a variety of transcription factors with tissue-specific effects; *n*-3 PUFA improve insulin metabolism by inhibiting pro-inflammatory cytokine release and NF-*κ*B protein expression^(^ ^194^ ^)^, and by activating their G-protein-coupled receptor 120^(^ ^195^ ^)^.

A recent meta-analysis showed that long-term supplementation of marine-derived *n*-3 PUFA results in a significant reduction of IL-6, TNF*α* and CRP plasma levels, in both healthy and non-healthy subjects^(^ ^196^ ^)^.

Moreover, the increasing vegetarian and vegan population requires alternative sources of LC-PUFA to guarantee them the appropriate intake of *n*-3 FA^(^ ^30^ ^,^ ^197^ ^,^ ^198^ ^)^. In addition to eating a larger quantity of vegetables, with a higher content of essential *n*-3 FA, a promising source of *n*-3 PUFA is represented by different species of microalgae^(^ ^199^ ^,^ ^200^ ^)^. To this purpose, the European Commission has authorised the use in certain foods of oil derived from microalgae *Schizochytrium* sp. rich in DHA and EPA (EU decision no. 2015/545 and no. 2015/546),

Altogether, these data provide useful scientific information for planning future investigations aimed at exploring PUFA effects when considering a nutritional therapy of inflammatory diseases and GDM.

## Conclusions and future research

GDM is increasing worldwide; obesity worsens this condition with increased risk of developing metabolic disorders in both mother and offspring later in life. The adoption of healthy lifestyle, with the adherence to a healthy dietary pattern, has positive effects on the prevention and management of diabetes. The Mediterranean diet is considered the model of healthy diet, and even more studies demonstrate its power in reducing the burden of several disorders in human being.

The few studies investigating the impact of healthy diet on GDM indicate that adherence to a healthy dietary pattern before pregnancy can reduce the risk of developing GDM; very few data about the association between dietary exposure and GDM outcomes are available.

Accordingly, this descriptive review tried to provide a picture on what we know about the impact of specific dietary biocompounds (i.e. polyphenols and PUFA) on the molecular mechanisms involved in glucose homoeostasis. Literature data, mostly derived from *in vitro* or animals studies, indicate that almost all subclasses of flavonoids, as well as the stilbene RSV and some olive oil phenolic compounds, interact and modulate several molecular pathways regulating insulin sensitivity in pancreatic *β*-cell, adipocyte, liver and muscle. Polyphenols drive activation and/ or silencing of transcription factors and consequently influence genes expression. Moreover, the fact that miRNA are the target of polyphenols action is to be taken into account in the future studies aimed at improving the understanding of the biological effects of polyphenols. However, how these compounds might work in GDM setting is a matter of speculation. Although increasing data indicate the beneficial effect of PUFA on pregnancy, a high-level evidence has not yet been achieved to make recommendations for GDM.

Many gaps, in the nutritional field and in the knowledge of pathophysiological processes, have to be filled in to progress in GDM prevention and management. GDM results from a complex interaction between the genetic background and environment. Identifying a number of modifiable biomarkers (including adipokines and miRNA) and the nutritional factors (e.g. polyphenols and their metabolites) able to target them can be useful in both early diagnosis and disease management.

It will be necessary to carry out dietary intervention studies involving large cohorts, and take into account genetic and dietary differences among races and countries. The ‘omics’ techniques will be fundamental tools to monitor dietary changes in populations and to identify the association between dietary exposure and disease outcomes. The integration of data from an individual’s genetic predisposition, endogenous metabolome, food metabolome and mirRNome profiles by systems biology approaches will provide a holistic view (systems biology approaches) of the relations between diet and disease, and will make it possible to translate these data in nutritional recommendations and diseases management. In particular, differences in metabolic profiles will be useful to identify novel biomarkers of dietary intake associated with disease. In this regard, the recent creation of a database to estimate intake and dietary seafood source of LC-PUFA in pregnancy provides a useful tool and represents an important step for future intervention studies.

Sharing this database along with others providing information on dietary polyphenols and polyphenol metabolites will help researchers to better understand the mechanisms governing interaction between dietary active compounds and the human organism.
